# Determinants of Facility-Level Use of Electronic Immunization Registries in Tanzania and Zambia: An Observational Analysis

**DOI:** 10.9745/GHSP-D-20-00134

**Published:** 2020-09-30

**Authors:** Emily Carnahan, Ellen Ferriss, Emily Beylerian, Francis Dien Mwansa, Ngwegwe Bulula, Dafrossa Lyimo, Anna Kalbarczyk, Alain B. Labrique, Laurie Werner, Jessica C. Shearer

**Affiliations:** a PATH, Seattle, WA, USA.; b Department of International Health, Johns Hopkins Bloomberg School of Public Health, Baltimore, MD, USA.; c National Expanded Programme on Immunisation, Ministry of Health, Lusaka, Zambia.; d Immunisation and Vaccines Development Program, Ministry of Health, Community Development, Gender, Elderly and Children, Dar es Salaam, Tanzania.

## Abstract

**Background:**

As more countries transition from paper-based to electronic immunization registries (EIRs) to collect and track individual immunization data, guidance is needed for successful adoption and use of these systems. Little research is available on the determinants of EIR use soon after introduction. This observational study assesses the determinants of facility health care workers’ use of new EIRs in Tanzania and Zambia, implemented during 2016 to 2018.

**Methods:**

We used EIR data entered between 2016 and 2018 from 3 regions in Tanzania and 1 province in Zambia to measure weekly EIR system use for a total of 50,639 facility-weeks. We joined secondary data on facility characteristics and applied the Performance of Routine Information System Management framework to categorize characteristics as organizational, technical, or behavioral. We used a generalized estimating equations logistic regression model to assess facility characteristics as potential determinants of system use.

**Results:**

In both countries, the estimated odds of weekly EIR use declined weekly after EIR introduction. In Tanzania, health centers and hospitals had increased odds of system use compared to dispensaries. For each additional health care worker trained in a facility during the EIR introduction, the estimated odds of weekly EIR use increased. Tanzanian facilities that had transitioned entirely to paperless reporting had higher odds of sustained use compared to those maintaining parallel electronic and paper-based reporting systems. In Zambia, distance from the district health office was significantly associated with decreasing odds of system use. There were significant differences in EIR use by district in both countries.

**Discussion:**

The results highlight the importance of organizational and behavioral factors in explaining sustained EIR use. As EIRs are introduced in new settings, we recommend indicators of engagement and use be built directly into the system so they can be routinely monitored, and course corrections can be implemented as needed.

## Abstract (precis)

We provide a framework to quantify the use of electronic immunization registry systems at the facility level and results show the importance of behavioral and organizational factors in explaining their sustained use in Tanzania and Zambia.

## Teaser

**Key Findings:**

- We provide a framework to quantify the facility-level use of electronic immunization registry (EIR) systems.
- Facility EIR use declined over time in all study regions in Tanzania and Zambia.
- There was greater EIR utilization among facilities at which health care workers reported into electronic systems only compared to parallel paper and electronic systems.

**Key Implications:**

- As more countries transition to EIRs, it is important to understand whether and why health care workers at facilities are adopting and using these systems.
- As EIRs are introduced in new settings, we recommend building indicators of engagement and use directly into the system for routine monitoring and implementing changes as needed.

## INTRODUCTION

Each year, vaccines prevent an estimated 2 to 3 million child deaths.^1^ Over the last 3 decades, vaccination coverage levels have significantly increased worldwide, but progress has stalled in recent years. In 2018, 19.4 million children did not receive the 3 recommended doses of diptheria, tetanus, and pertussis vaccine in their first year of life.^1^ In low- and middle-income countries (LMICs), efforts to strengthen vaccination coverage and equity are often impeded by inaccurate or incomplete data on routine childhood immunizations.^2^ As many country health systems are integrating new digital tools, there is an opportunity to use digital tools to improve immunization programs’ pursuit to close the vaccination gap.^3^

The electronic immunization registry (EIR) is one such digital tool to support immunization program performance. EIRs are routine systems to capture, store, access, and share individual, longitudinal health information in digitized records.^4^ They focus on collecting individual immunization records but can also collect other health and demographic data in each individual’s digital record and can be linked with vaccine stock management, human resources management, or other health management information systems. There is promising—although limited—evidence that digital health tools can improve vaccination adherence, uptake, and the efficiency of immunization programs.^5^ In high-income countries, where they are routinely used, EIRs have been shown to support tracking individual vaccine eligibility and delivery, facilitate vaccine management and accountability, and inform assessments of vaccination coverage, missed opportunities, and disparities, among other benefits.^6^ Thus, EIRs have been proposed as a solution to improve data quality, facilitate reporting, and promote data use in LMICs, ultimately providing the opportunity to strengthen vaccination services.^7^

Traditionally, most LMICs have relied on paper-based data collection at the facility level to capture immunization data.^3^^,^^8^^,^^9^ As country demand to integrate digital solutions to improve health outcomes is increasing,^10^ there are also more examples of LMICs developing, implementing, and scaling EIRs across many countries in Latin America, Africa, and Asia.^11^^–^^14^ The Bill and Melinda Gates Foundation, Gavi, and other funders have invested substantially in EIRs in LMICs to improve immunization data quality and use to increase vaccine coverage and equity. However, few EIR implementations in LMICs have been rigorously evaluated.^9^ And despite improved political, financial, and technical support to introduce digital health strategies, many of these interventions continue to face barriers to adoption and consistent use that limit their potential impact.^3^^,^^15^^–^^18^

As more countries transition from traditional systems using paper-based data collection tools to EIRs, guidance is needed to ensure the ongoing use of these new systems. This study uses an implementation science framework to test hypotheses of the drivers of EIR use. Understanding the drivers can inform improvements to the system design and/or implementation strategies for more effective implementation and sustained use. This article seeks to identify determinants of facility-level differences in system use among facilities in Tanzania and Zambia where EIRs have been implemented.

We used an implementation science framework to identify determinants of EIR use in facilities in Tanzania and Zambia.

## THE EIR INTERVENTION AS PART OF TANZANIA AND ZAMBIA’S EHEALTH LANDSCAPE

Tanzania and Zambia are the focus of this study based on their role as demonstration countries in the BID Initiative. Funded by the Bill and Melinda Gates Foundation, the BID Initiative is grounded in the belief that better data plus better decisions will lead to better health outcomes.

In Tanzania and Zambia, facility health care workers (HCWs) use paper-based tools to capture information about vaccine delivery and stock.^19^^,^^20^ Each facility manually aggregates the data into a monthly report that is submitted to the district; from there, data are entered into electronic systems and aggregated to the district, regional, and national levels. Beginning in 2013, the Tanzanian and Zambian Ministries of Health (MOHs), in partnership with PATH, identified challenges related to immunization data quality and use and then iteratively developed solutions to address them.

The most pressing immunization data quality and use challenges (categorized by the World Health Organization Classification of Digital Health Interventions health system challenges^4^) included the following:

- Incomplete or untimely data (1.3)
- Inaccurate or uncertain population denominators to inform coverage calculations (1.1)
- Lack of unique identifiers for infants (1.7)
- Difficulty identifying children who do not start immunization or who drop out (defaulter tracing) (1.5, 8.6)
- Poor data visibility at the facility level (1.5)
- Complex data collection tools
- Insufficient data on supply chains and logistics management (1.3)
- Inadequate capacity for data management and use (1.6)

The BID Initiative intervention strategy to address these challenges included both technological and change management components to foster an environment conducive to data use for decision making.^21^ The package of solutions included the development of a standards-based EIR with automated, simplified reports; web-based dashboards; and supply chain system tools,^12^ and the introduction of data use mentors, peer networking communication forums between health workers, and data use guides to build capacity and motivation for data use.^22^ Interventions were designed according to the principles of user-centered design to address each country’s identified challenges.^23^^,^^24^ For example, in both countries, the MOHs codesigned requirements for the EIRs with the project team, and user advisory groups (comprised of HCWs from facility, district, and regional levels) tested iterations of the EIRs to provide feedback on the functionality and user interface.^12^

The BID team tested and refined interventions in pilot facilities in each country before scaling up a package of interventions to other facilities. The interventions were introduced to HCWs through on-the-job training with staff from higher levels of the health system (district, regional, national) engaged to provide a supportive environment through championing data use practices, mentoring facility staff, and holding facilities accountable for their performance.^25^

## DETERMINANTS OF EIR USE: A CONCEPTUAL FRAMEWORK

We sought to evaluate facility characteristics associated with EIR use following the rollout of interventions in the first regions of implementation—Arusha, Kilimanjaro, and Tanga regions, Tanzania, and South-ern Province, Zambia—from 2016 to 2018. The successful adoption of EIRs requires many organizational, technical, and behavioral factors to come together, as conceptualized in the Performance of Routine Information System Management framework.^26^ Facility characteristics in this study were mapped to these categories of determinants. The hypothesized impact of these characteristics on EIR use are shown in Table 1 and described here.

**TABLE 1.** Hypotheses on Impact of Facility Characteristics on EIR Use Aligned to PRISM Framework

**PRISM Framework Determinant**	**Variable**	**Hypotheses**
Organizational	Paperless reporting	If a facility transitions to paperless reporting (only using the EIR as the official system), it will be more likely to use the EIR.
	Facility volume	If a facility has a larger patient population, HCWs may have a busier daily patient load, therefore less time for data entry and will be less likely to use the EIR. OR, if a facility has a larger patient population, HCWs may see more value in using the EIR to manage their patient population and will be more likely to use the EIR.
	Facility type	If a facility is a hospital or health center, it may have more resources (e.g., equipment, skilled HCWs) compared to a dispensary and may be more likely to use the EIR.
	Ownership type	If a facility is public, HCWs may feel greater ownership of the decision to adopt the EIR and/or feel more accountable to use the EIR than in private facilities and thus may be more likely to use the EIR.
	Distance to district health office	If a facility is located closer to the district health office, it will be more likely to receive in-person support from district health officials.
	Training strategy (Tanzania only)	If a facility received the second training strategy (i.e., district staff provided additional support and training), it will be more likely to use the EIR than facilities who received the first training strategy, which relied on BID project staff.
	Number of immunization sessions per week	If a facility provides more immunization sessions per week, they will be more likely to enter data into the EIR each week.
Technical	Primary power source	If a facility has a consistent electricity connection, it will be more likely to use the EIR.
	Internet connectivity	If a facility has a consistent internet connection, it will be more likely to use the EIR.
Behavioral	Number of HCWs trained per facility	If a facility has more HCWs trained, it will be more likely to use the EIR.
	Weeks since EIR introduction	As the length of time since EIR introduction increases, facilities will be less likely to use the EIR.

Abbreviations: EIR, electronic immunization registry; HCW, health care worker; PRISM, Performance of Routine Information System Management.

### Organizational Factors

Organizational factors included the level of supervisory and political support for the new system, availability of human and financial resources, and management support. These factors were manifested at the facility, district, or regional level through informal norms, values, and practices or through formal guidelines, standards, and policies. As EIRs were introduced, organizational policies often required HCWs to continue the traditional paper-based data entry in addition to entering data electronically; this was the case in Tanzania and Zambia where HCWs were expected to conduct parallel data entry in the EIR and official paper-based reporting system until officially switching to paperless reporting. We hypothesized that when a facility transitioned to paperless reporting (i.e., only using the EIR), system use would have increased. A study evaluating SmartCare EIR use in Zambia observed parallel data entry requirements undermined SmartCare EIR adoption by clinic staff.^27^ We hypothesized based on implementation experience that HCWs in facilities responsible for a larger population would have had a busier daily client load, therefore less time for data entry. A study of the Mobile Technology for Health program in Ghana found that higher volume health centers and hospitals were less likely than community-based facilities to register and upload individual-level health information to the mobile platform, in part due to overburdened HCWs.^28^ However, we also hypothesized the reverse could be true: that HCWs in facilities with a larger patient population may have seen more value in using the EIR to manage their patients and would have been more likely to use the system. We also hypothesized relationships between EIR use and facility type, ownership, distance to the district health office (DHO) (as a proxy for supportive supervision), training strategy, and number of immunization sessions offered per week (Table 1).

### Technical Factors

Technical factors, such as user-interface design and offline functionality, were likely to affect the user’s experience with an EIR system as well as the system’s feasibility and acceptability. Considering that the system itself was the same across facilities, we hypothesized that facilities with a consistent connection to internet and electricity would have been more likely to use the system. Other studies have shown that EIR adoption in 2 districts in Uganda was impeded by blackout days (no electricity or internet connectivity)^29^ and that power outages (“load-shedding” or brown-outs) were the primary challenge to using the SmartCare electronic health record system for immunization data in Zambia.^27^ Technical factors could have affected system performance directly or mediated through behavioral factors. For example, in Zambia, clinic staff’s experience with failed attempts using SmartCare and concerns regarding data loss impaired EIR uptake.^27^

### Behavioral Factors

Behavioral factors, such as HCWs’ capability and motivation to use the new system, required careful attention during system design, implementation, and beyond. We hypothesized that HCWs who received adequate training and more or higher quality ongoing supportive supervision would have been more likely to use the EIR. A review of an EIR introduction in Uganda highlighted the importance of onsite technical support and on-the-job training.^29^ We hypothesized that if more HCWs in a facility had been trained to use the EIR, the facility would have been more likely to have sustained use of the EIR over time. We also hypothesized that HCW motivation to use the EIR would possibly wane over time, so as the length of time since EIR introduction increased, facilities would have been less likely to use the EIR. A recent systematic review of data use interventions found that HCWs were not motivated to adopt or use new digital interventions when they replaced a status quo that was perceived to work adequately.^9^ Others have suggested the perceived threat of increased data transparency (and thereby potential scrutiny) may limit enthusiasm for digital systems.^30^ Although other behavioral factors (e.g., HCWs’ attitudes, skills, and motivation) are important determinants, this study was limited to including behavioral factors that could be measured using existing secondary data sources.

### Additional Factors

Other factors, such as national leadership, governance, and policy, are important aspects of the enabling environment for any digital tool^30^^,^^31^; however, they were not included in this study due to the focus on determinants at the facility level.

## METHODS

### Study Setting

The study data are from electronic vaccine records in 3 regions of Tanzania: Arusha, Kilimanjaro, and Tanga, and in Zambia’s Southern Province, where the BID Initiative interventions were initially implemented. The regions in Tanzania were chosen by the government as the first implementation regions due to their nomadic communities and porous borders with Kenya, which posed challenges to estimating population denominators for monitoring immunization coverage. Southern Province in Zambia had similar challenges due to sharing a border with Zimbabwe in the southwest and Botswana in the southeast. The regions in Tanzania and Zambia were also chosen for their mix of urban, peri-urban, and rural communities. Moreover, Southern Province in Zambia was selected as the pilot province due to underperformance of the immunization program and strong commitment from the district and provincial leaders.

The 3 regions in Tanzania collectively have a population of 5.38 million, cover a land mass of over 77,500 square kilometers, and support 847 health facilities that provide immunization services.^32^^–^^34^ Southern Province in Zambia has a population of 1.96 million, covers a land mass of 85,283 square kilometers, and supports 298 health facilities that provide immunization services.^35^

### Data

We used data routinely collected through the EIRs in Tanzania and Zambia. Tanzania first introduced a system called the Tanzania Immunization Inform-ation System in Arusha region in 2016–2017, which was later replaced with an improved system called the Tanzania Immunization Registry (TImR) built upon the OpenIZ platform (now known as SanteDB).^12^^,^^36^ Rollout was phased across 26 districts beginning in the Tanga, Arusha, and Kilimanjaro regions. Zambia introduced a system called the Zambia Electronic Immunization Registry (ZEIR) that was built upon the OpenSRP platform,^12^^,^^37^ beginning in Southern Province with a phased rollout across the 13 districts. TImR and ZEIR are both open-source software developed for use on tablets with online and offline functionality. In both countries, paper-based reporting remained the official system for capturing childhood immunization status until facilities fully transitioned to paperless reporting.

All data collected via Tanzania and Zambia’s routine immunization programs were released for analysis with permission from the Governments of Tanzania and Zambia. Institutional review board approval was obtained from both countries and a non-human subjects research determination was received from the PATH institutional review board. The analysis team received anonymized digital data. Data were processed and analyzed using Tableau, Alteryx, R, and STATA.

Data from the 3 regions in Tanzania were extracted from TImR on June 11, 2018, and data from Southern Province in Zambia were extracted from ZEIR on October 3, 2018. We excluded data for the final (partial) month and penultimate month to avoid completeness issues as a result of facilities working in the offline mode that had not yet synced their data for the most recent months. We included all facilities that had ever entered data into the EIR, thereby excluding facilities that did not provide immunizations. Table 2 provides descriptive information about the EIR datasets. Each data record was a single service delivered to an individual, and an individual could have had multiple services delivered during a single visit. Services included child weights captured, child or adult vaccines delivered, and nonvaccine interventions delivered (e.g., vitamin A, mebendazole, or insecticide-treated nets).

**TABLE 2.** Description of the Datasets Extracted From the Tanzania Immunization Registry and Zambia Electronic Immunization Registry

	**Tanzania**			**Zambia**
	**Arusha Region**	**Kilimanjaro Region**	**Tanga Region**	**Southern Province**
Number of districts	6	6	8	13
Number of facilities	283	292	330	551 (static and outreach sites)
Number of unique individuals	137,130	35,084	89,740	96,617
Number of records	1,606,776	206,871	671,562	1,323,264
Date range of EIR records (including back-entered data)	January 2015 – April 2018	January 2015 – April 2018	January 2015 – April 2018	January 2015 – August 2018
Date range of EIR introduction	June 2016 – March 2017	December 2017 – February 2018	July 2017 – August 2017	July 2017 – March 2018

Abbreviation: EIR, electronic immunization registry.

Datasets were cleaned for analysis to exclude historical records (back-entered data) by only considering data entries with a date after the EIR had been introduced in each facility. This eliminated the effect of large batches of backlogged records submitted by individual facilities shortly after the EIR deployment.

Secondary data on facility characteristics were collected by the BID Initiative implementation team during EIR rollout. Facility types and ownership were categorized based on government definitions. Distance from the DHO was calculated using facility GPS coordinates. Data on facility volume were extracted from government health management information systems.

### Measures of System Use

We used the dates of data entry into the EIR as a proxy for system use. We measured system use on a weekly basis for each facility based on whether there was any EIR activity at that facility, as measured by whether data records capturing any events (e.g., vaccine dose delivered, child weight recorded) were entered into the EIR for the given week. Our outcome variable for system use was binary, with 1 indicating that at least 1 data record had been entered into the EIR for that facility-week.

### Analysis

We used generalized estimating equation logistic regression models (xtgee in STATA with AR[1] correlation structure) to obtain marginal estimates of the odds ratios associated with facility characteristics and to account for within-facility correlation arising from longitudinal panel data with multiple observations (weeks) for each facility (the panel identifier). Covariates were chosen based on our conceptual framework, existing literature, and the authors’ firsthand experience participating in the BID Initiative implementation.

Organizational covariates included the facility type, ownership (public or private), volume of service delivery (number of vaccines administered monthly in Tanzania and annual number in attendance at child health clinics in Zambia), number of immunization sessions provided per week (Zambia only), and an indicator for whether the facility had transitioned to paperless reporting (Tanzania only). Distance from the DHO was included as a proxy for support from district health officials, as we hypothesized that they were more likely to provide in-person support to facilities that are physically closer. The training strategy provided in Tanzania was also tested as an organizational covariate, as midway through implementation in the first region, the strategy shifted from on-the-job training provided by BID project staff to leveraging district staff to provide additional support and training. Technical covariates included the facility’s primary power source and internet connectivity. Behavioral covariates included the number of HCWs initially trained during implementation rollout and the total number of weeks since the EIR was introduced at the facility. The data sources for covariates included facility characteristics captured in the EIRs, BID Initiative program data, government administrative data, and historical vaccine delivery data from paper-based sources.

Results were modelled separately for each country using complete case analysis. Covariates were first tested for a bivariate significant relationship with the outcome variable, and those with statistical significance were included in the final regression model. The final model for Tanzania included time since EIR introduction, facility type, number of HCWs trained on the EIR, and an indicator for when the facility transitioned to paperless reporting as covariates. The final model for Zambia included time since EIR introduction, distance from the DHO, and the number of immunization sessions provided per week. The final models for both countries additionally included the district where the facility was located. We hypothesized system use would differ by district due to a host of organizational, technical, and behavioral factors, both captured and not captured in the final models. Districts were included to control for confounding between the previously mentioned covariates and EIR use resulting from unmeasured facility characteristics imparted by the district in which the facility was located. The district that was expected to demonstrate the highest performance was selected as the comparator group in each model. In Tanzania, district-level units included city councils (CC), district councils (DC), municipal councils (MC), or town councils (TC). For the Tanzania model, Arusha CC in Arusha region was selected, as it was the pilot implementation district, thus had received additional support, and includes the capital and largest city in Arusha region. For the Zambia model, Choma district was selected as it is the capital district for Southern Province.

## RESULTS

### Activity

**FIGURE 1.**
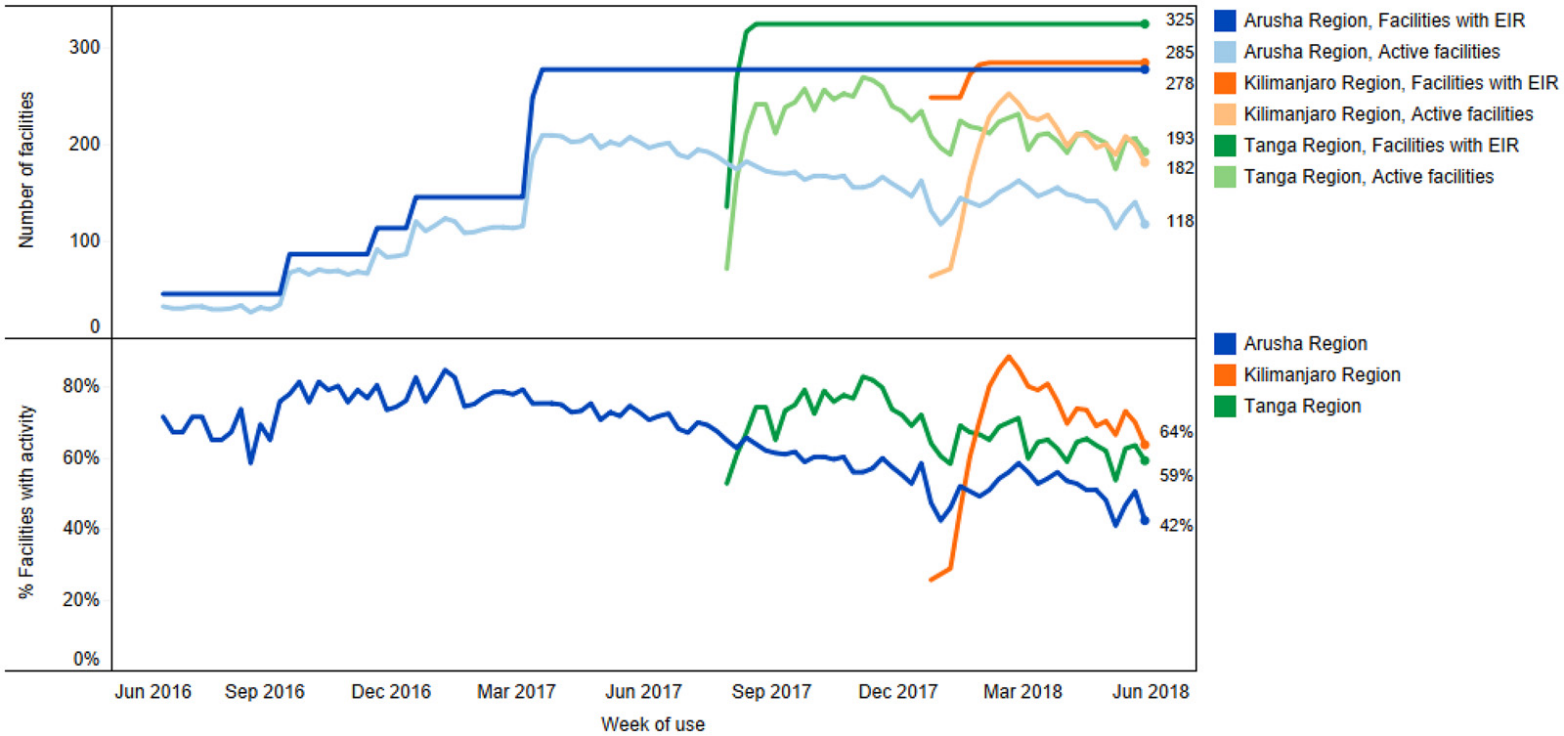
Number of Facilities in Tanzania Using the EIR (top) and Percentage of Facilities Using the EIR (bottom) Abbreviation: EIR, electronic immunization registry.

**FIGURE 2.**
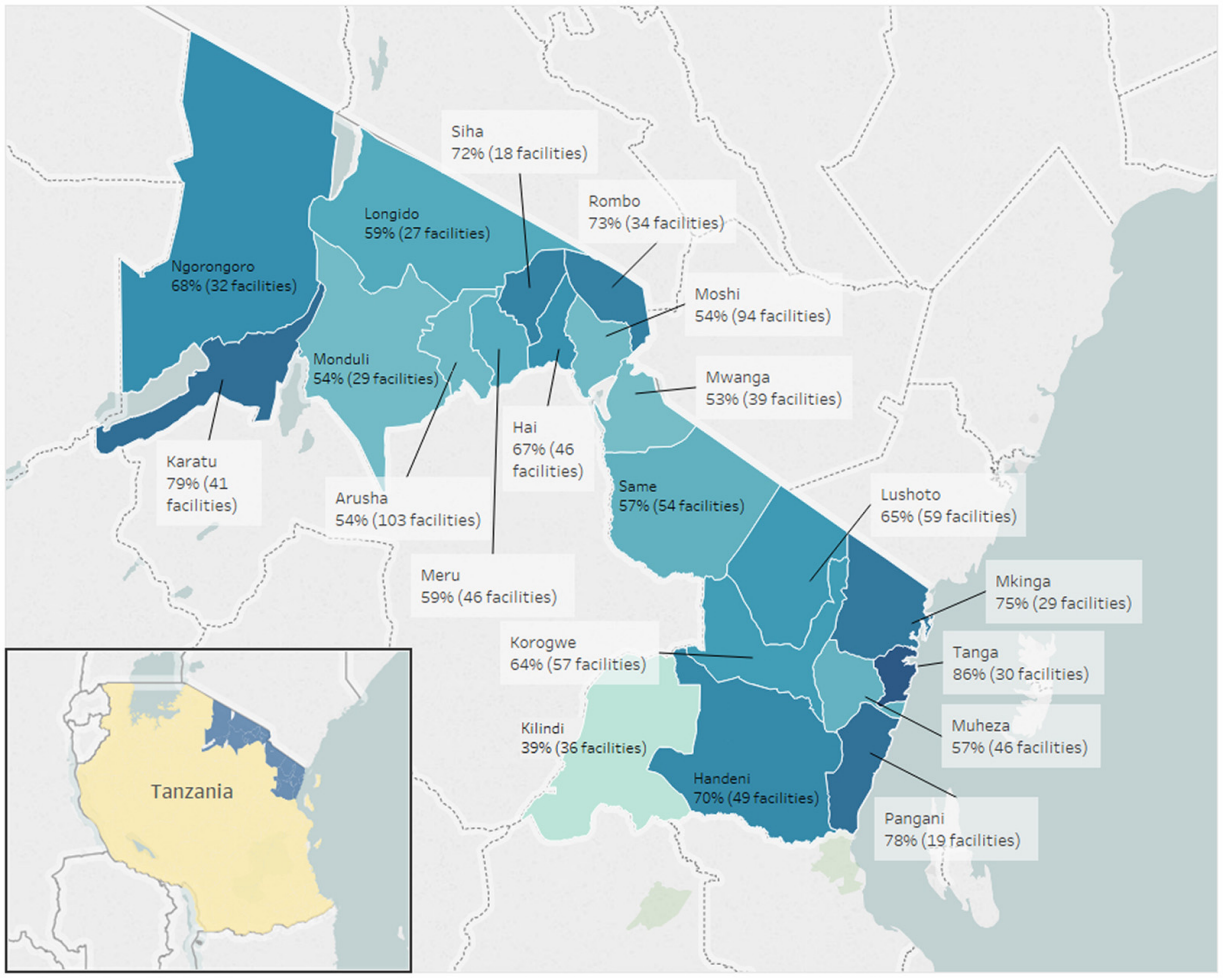
Facility Average Percentage of Active Weeks of EIR Use by District, Tanzania, 2016–2018 Abbreviation: EIR, electronic immunization registry.

In Tanzania, the package of interventions was implemented in 285 facilities in Arusha, 327 facilities in Tanga, and 312 facilities in Kilimanjaro region. Figure 1 shows the cumulative number of facilities using the EIR based on staged introduction, and of those facilities, the percentage that used the system in each week. In Arusha region, the number of facilities where the EIR had been introduced gradually increased from June 2016 to March 2017 as this was the pilot region where interventions were being iteratively adapted and gradually introduced to new districts, ultimately resulting in 278 unique facilities using the system. There was a much more rapid scale-up in Kilimanjaro and Tanga regions, where interventions were introduced across all districts within 2–3 months. In Tanga region, 325 facilities had ever entered data into TImR, and in Kilimanjaro, 285 facilities, similarly, had entered any data. Across all regions, the number of active facilities plateaued and then declined over time. We calculated the number of active weeks as a percent of total weeks since EIR introduction by facility; Figure 2 shows the facility average by district in Tanzania. The district average ranges from 39% in Kilindi (meaning on average, facilities in Kilindi used the EIR for 39% of the total weeks since introduction) to 86% in Tanga district within the Tanga region.

**FIGURE 3.**
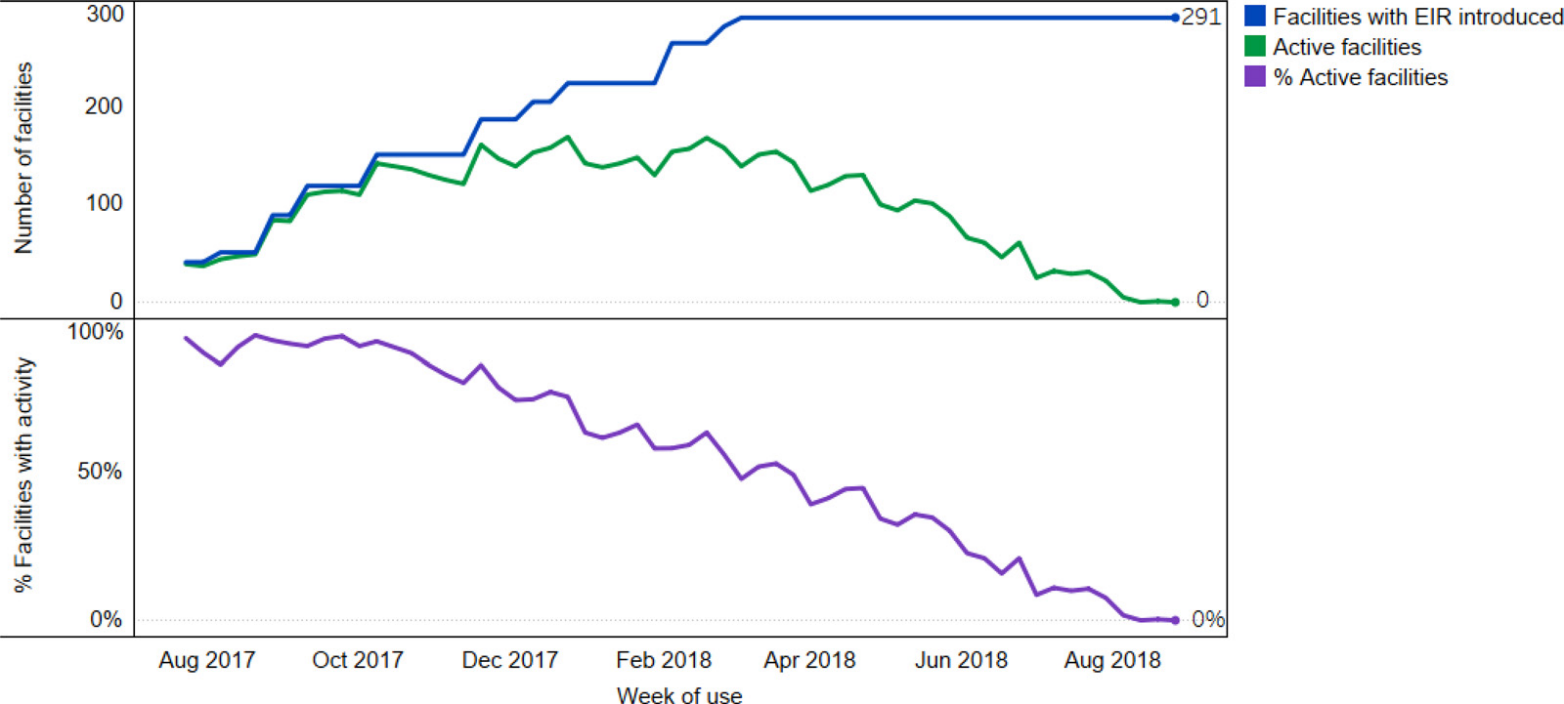
Number of Facilities in Southern Province, Zambia Using the EIR (top) and Percentage of Facilities Using the EIR (bottom) Abbreviation: EIR, electronic immunization registry.

**FIGURE 4.**
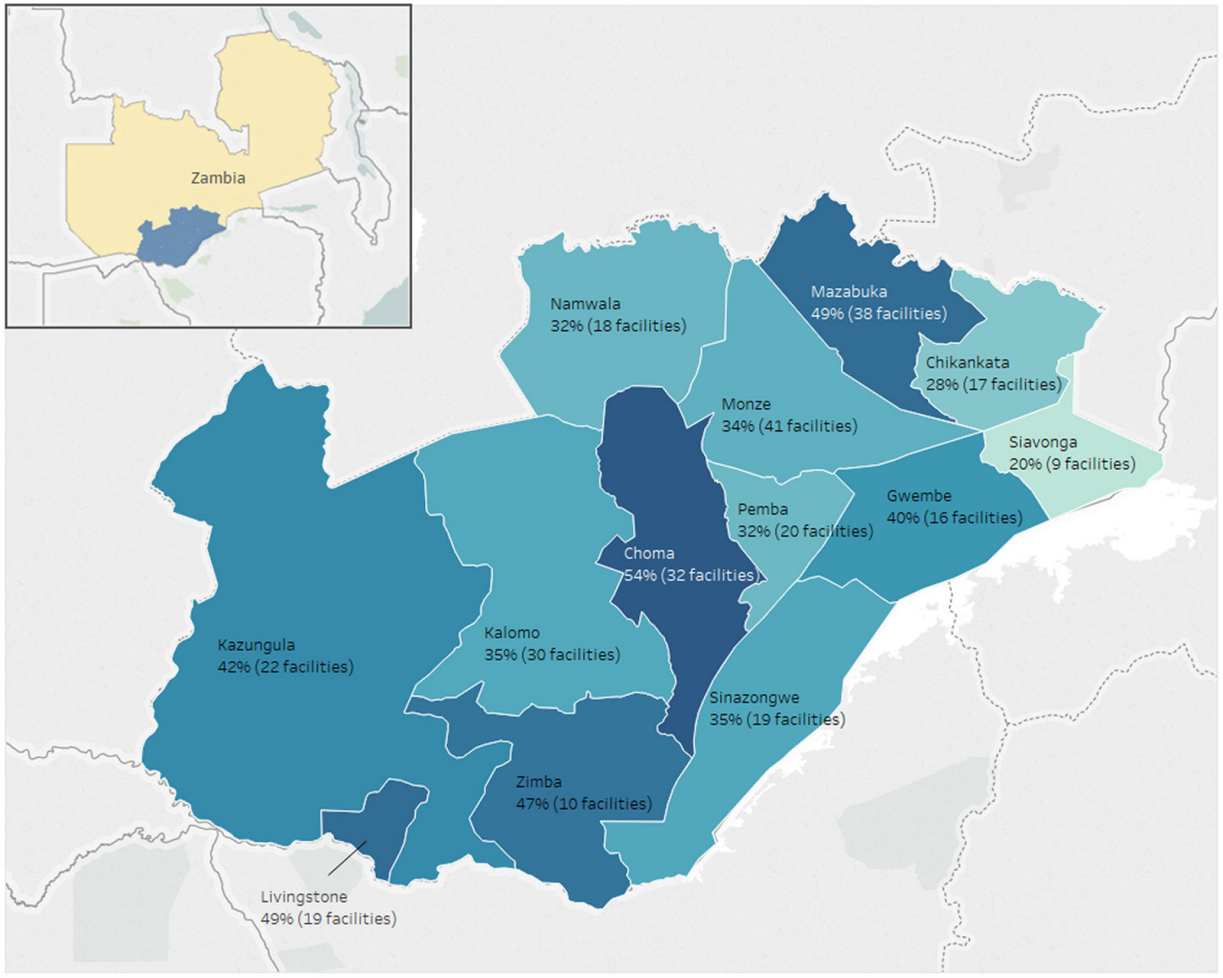
Facility Average Percentage of Active Weeks of EIR Use by District, Southern Province, Zambia, 2017–2018 Abbreviation: EIR, electronic immunization registry.

In Zambia, the package of interventions was introduced in 298 facilities across 13 districts in Southern Province beginning in November 2016 in Livingstone District. ZEIR was first introduced in Livingstone and Kazungula Districts in July 2017 and was scaled up to the other districts through March 2018. Figure 3 shows the number of active facilities over time as the EIR was introduced. Like Tanzania, there was a gradual increase in use as the EIR was introduced to new districts, but then system use began to decline over time. The steady decline in EIR use was similar across districts (results not shown). In August 2018, fewer than 5% of facilities where the EIR had been introduced in Southern Province had entered data. Figure 4 shows the facility average percentage of weeks active in the EIR by district. The district average ranges from 20% in Siavonga to 54% in Choma.

### Facility Determinants of Weekly Activity

The regression models (1 per country) explain the relative contribution of the facility- and district-level determinants associated with weekly EIR use. Table 3 describes the independent variables that were tested for inclusion in the models, and Tables 4 and 5 show the model results for each country.

**TABLE 3.** Description of the Facility Characteristics Included in the Regression Models for EIR Use, Tanzania and Zambia

	**Tanzania**				**Zambia**
	**Arusha**	**Kilimanjaro**	**Tanga**	**All Regions**	**Southern** **Province**
Number of districts	6	6	8	20	13
Number of facilities	278	285	326	889	282
**Organizational**					
Paper-based records	100.0%	100.0%	89.9%	96.3%	100.0%
Paperless records	0.0%	0.0%	10.1%	3.7%	0.0%
Number of monthly vaccine doses delivered, mean (SD)	341.3 (529.1)	206.2 (246.5)	323.3 (314.3)	288.8 (372.5)	–
Annual child health clinic attendance, mean (SD)	–	–	–	–	5351.7 (4220.5)
Facility type					
Dispensary	78.4%	79.3%	86.5%	81.7%	0.0%
Health center	16.9%	15.8%	0.0%	10.3%	76.1%
Hospital	4.7%	4.9%	2.5%	3.9%	1.8%
Hospital affiliated center	0.0%	0.0%	0.0%	0.0%	4.4%
Missing	0.0%	0.0%	11.0%	4.0%	17.6%
Ownership type					
Private	32.4%	24.6%	12.0%	22.4%	–
Public	65.1%	70.5%	85.6%	74.4%	–
Missing	2.5%	4.9%	2.5%	3.3%	–
Distance to DHO, km, mean (SD)	37.2 (31.0)	61.8 (171.5)	23.4 (14.4)	35.9 (68.8)	46.7 (39.7)
On-the-job training by BID Initiative staff	71.6%	0.0%	0.0%	22.4%	–
Additional support and training by district staff	28.4%	100.0%	100.0%	77.6%	–
Number of immunization sessions per week					
1 or more	–	–	–	–	77.4%
Less than 1	–	–	–	–	11.0%
Missing information	–	–	–	–	11.6%
**Technical**					
Primary power source					
Grid	36.7%	79.3%	0.0%	36.9%	43.6%
Solar	31.3%	7.7%	0.0%	12.3%	1.8%
None	4.0%	0.0%	0.0%	1.2%	51.1%
Missing	28.1%	13.0%	100%	49.6%	3.5%
Internet connectivity					
Yes	60.8%	–	–	–	–
No	6.8%	–	–	–	–
Missing	32.4%	–	–	–	–
**Behavioral**					
Number of HCWs trained per facility, mean (SD)	2.2 (0.9)	2.1 (1.2)	2.5 (1.1)	2.3 (1.1)	–

Abbreviations: DHO, district health office; EIR, electronic immunization registry; HCW, health care worker; SD standard deviation.

**TABLE 4.** Results of Regression Model Predicting EIR Use for Facilities in Tanzania

**Variable**	**Estimate (odds ratio)**	**Robust Standard Error**	***P*-Value**
**Organizational**			
Paperless (compared to using parallel systems)^a^	2.72	0.83	.001
Facility Type (compared to dispensary)			
Health center^a^	1.61	0.33	.02
Hospital^a^	3.82	1.13	<.001
**Behavioral**			
Number of HCWs trained^a^	1.35	0.09	<.001
Weeks since EIR introduction^a^	0.98	<0.01	<.001
District (Region)^b^			
Tanga CC (Tanga)^a^	2.89	1.07	.004
Karatu DC (Arusha)^a^	2.45	0.81	.007
Mkinga DC (Tanga)	1.83	0.64	.09
Pangani DC (Tanga)	1.56	0.54	.20
Longido DC (Arusha)	1.53	0.65	.32
Ngorongoro DC (Arusha)	1.53	0.56	.24
Handeni TC (Tanga)	1.32	0.70	.60
Korogwe TC (Tanga)	1.19	0.62	.74
Siha DC (Kilimanjaro)	1.12	0.50	.80
Meru DC (Arusha)	1.11	0.43	.79
Handeni DC (Tanga)	1.05	0.46	.92
Monduli DC (Arusha)	1.00	0.54	.99
Rombo DC (Kilimanjaro)	0.99	0.34	.97
Arusha DC (Arusha)	0.90	0.45	.84
Bumbuli DC (Tanga)	0.82	0.31	.61
Korogwe DC (Tanga)	0.70	0.23	.30
Lushoto DC (Tanga)	0.65	0.22	.20
Moshi MC (Kilimanjaro)	0.62	0.24	.22
Hai DC (Kilimanjaro)	0.58	0.20	.18
Mwanga DC (Kilimanjaro)	0.53	0.20	.10
Same DC (Kilimanjaro)^a^	0.51	0.17	.05
Muheza DC (Tanga)^a^	0.49	0.17	.04
Kilindi DC (Tanga)^a^	0.35	0.13	.005
Moshi DC (Kilimanjaro)^a^	0.29	0.09	<.001

Abbreviations: CC, city council; DC, district council; EIR, electronic immunization registry; HCW, health care worker; MC, municipal council; TC, town council.

a Statistically significant at alpha=.05 level.

b Compared to Arusha city council, which was selected as it was the pilot implementation district and contains the capital and largest city in Arusha region.

**TABLE 5.** Results of Regression Model Predicting EIR Use for Facilities in Zambia

**Variable**	**Estimate (odds ratio)**	**Robust Standard Error**	***P*-Value**
**Organizational**			
Less than 1 immunization day/week	0.82	0.31	.60
Distance from DHO, compared to 1^st^ quartile			
2^nd^ quartile^a^	0.46	0.15	.015
3^rd^ quartile^a^	0.41	0.14	.007
4^th^ quartile^a^	0.32	0.11	.001
**Behavioral**			
Weeks since EIR introduction^a^	0.88	<0.01	<.001
District^b^			
Zimba	1.16	0.50	.74
Kazungula	0.97	0.48	.95
Mazabuka	0.91	0.40	.83
Livingstone	0.73	0.40	.57
Gwembe^a^	0.33	0.17	.03
Kalomo^a^	0.30	0.12	.003
Namwala^a^	0.19	0.08	<.001
Sinazongwe^a^	0.14	0.08	.001
Pemba^a^	0.10	0.04	<.001
Monze^a^	0.08	0.03	<.001
Chikankata^a^	0.05	0.03	<.001
Siavonga^a^	0.03	0.03	<.001

Abbreviations: DHO, district health office, EIR, electronic immunization registry.

a Statistically significant at alpha=.05 level.

b Compared to Choma district, which was selected as it is the capital district for Southern Province and expected to be a high performer.

#### Tanzania

In Tanzania, 2 organizational determinants, facility type and whether the facility had transitioned fully to paperless reporting were significant predictors of EIR use (Table 4). Compared to dispensaries, health centers were 61% more likely (odds ratio [OR]=1.61; 95% confidence interval [CI]=1.08, 2.42) to use the system, while hospital odds of use were 3.83 (95% CI=2.14, 6.85) times greater. Facilities that had transitioned to completely paperless reporting had odds of weekly EIR use that were 2.76 (95% CI=1.54, 4.94) times as large as facilities using parallel EIR and paper reporting systems. The log-transformed median number of doses delivered per month, a measure of facility volume, was found to be significantly associated with EIR use in the bivariate model (OR=1.76; 95% CI=1.57, 1.98); however, this was excluded from the final model due to collinearity with facility type and number of health workers trained. Ownership type, distance to the DHO, and the training strategy received, were not found to be significantly associated with EIR use in bivariate analyses, thus were excluded.

Technical determinants of EIR use were not included in the final model. In bivariate analysis, facilities with no primary power source were estimated to have significantly lower weekly EIR use compared to facilities that were connected to the electric grid (OR=0.35; 95% CI=0.15, 0.81). However, primary power source was excluded from the final model as data were not available for Tanga region. Internet connectivity was not significantly associated with system use in bivariate analysis, thus was also excluded.

Behavioral determinants significantly associated with EIR use included the number of HCWs trained and weeks since EIR introduction. For each additional HCW that was trained during the EIR introduction, estimated odds of weekly EIR use were 1.39 (95% CI=1.22, 1.58) times greater. For each additional week from EIR introduction, the odds of use were 1.9% lower (95% CI=1.5%, 2.3%) (OR=0.98; 95% CI=0.98, 0.99).

Facilities in most districts did not have significantly different odds of using the EIR compared to facilities in Arusha CC. The exceptions were facilities in Tanga CC and Karatu DC, which were estimated to have significantly higher odds of use compared to facilities in Arusha CC, and facilities in Same DC, Muheza DC, Kilindi DC, and Moshi DC, which were estimated to have significantly lower odds of use compared to facilities in Arusha CC.

#### Zambia

In Zambia, distance from the DHO was the only organizational determinant significantly associated with EIR use (Table 5). Facilities in the second distance quartile were estimated to have 0.46 times (95% CI=0.24, 0.86) the odds of weekly use compared to facilities in the first quartile. Facilities in the third and fourth quartiles were estimated to have 0.41 (95% CI=0.21, 0.79) and 0.32 times (95% CI=0.17, 0.63) the odds of weekly use compared to facilities in the first quartile for distance to the DHO. Urban/rural status was significantly associated with EIR use, however was collinear with distance to the DHO, thus excluded from the final model. Odds of use were not significantly different between facilities offering vaccination days less than once a week and those offering vaccination days at least once a week. Facility volume (measured as 2017 attendance at child health clinics) and facility type were not found to be significantly associated with EIR use in bivariate analyses, thus were excluded from the model.

The only technical determinant assessed for Zambia, primary power source, was not found to be statistically significantly associated with EIR use, thus was omitted from the final model.

The only behavioral covariate significantly associated with EIR use was time since EIR introduction. The odds of EIR use decreased by 12.4% (95% CI=11.5, 13.4) per week from introduction.

Districts with significantly lower odds of estimated weekly EIR use included Gwembe, Kalomo, Namwala, Sinazongwe, Pemba, Monze, Chikankata, and Siavonga. Odds of use were not significantly different in Zimba, Kazungula, Mazabuka, or Livingstone districts compared to Choma.

## DISCUSSION

The descriptive analyses based on facility EIR data showed declines in weekly EIR use post-introduction across all regions in Tanzania and Zambia. The statistical analyses joined the EIR data with secondary data on other facility characteristics to explain which facility determinants were associated with system use.

Organizational factors that were strongly associated with weekly EIR use were facility type and paperless reporting in Tanzania and distance to the DHO in Zambia. The results show higher odds of system use associated with hospitals and health centers compared to dispensaries in Tanzania. This may have been because larger facilities were more likely to have adequate HCWs dedicated to immunization of whom some could prioritize data entry, as opposed to dispensaries where a single HCW would likely be stretched across functional areas. HCWs in hospitals or health centers may have had greater technical skills, training, and capacity compared to HCWs in dispensaries so they may have been more adept at using the system. HCWs in larger facilities may have also perceived the EIR as more valuable to support their day-to-day work to manage and track a large client population—a task that would be more manageable with paper-based forms if the client population was smaller.

Facilities in Tanzania that had transitioned to paperless reporting were significantly more likely to use the EIR in a given week compared to facilities that were still responsible for parallel systems. BID staff in both countries observed that when HCWs had limited bandwidth, they prioritized data entry in the paper-based tools (the official reporting system) over using the EIR. Once a facility transitioned to using the EIR as their official tool, the odds of system use were much higher. In Tanzania, a national assessment in collaboration with the regional office in Tanga identified 33 facilities that transitioned to paperless reporting in March 2018; these facilities were selected to represent a mix of high and low performers with representation from all the district councils in Tanga. Since then (and not captured in our analysis), an additional 2 districts (60 facilities) in Tanga region transitioned to paperless reporting in September 2018. The Tanzania MOH and the President’s Office, Regional Administration and Local Govern-ment developed a checklist of criteria to inform the decision to transition facilities to paperless reporting. As more facilities migrate to paperless reporting, there is an opportunity to continue to test our hypothesis and confirm the results presented here that show paperless reporting increases system use.

Facilities in Tanzania that had transitioned to paperless reporting were significantly more likely to use the EIR compared to facilities that were still responsible for parallel systems.

In Zambia, those facilities that were farther from the DHO had significantly lower odds of using the system. Our initial hypothesis was that those farther facilities may have been less likely to have received supportive supervision or other district support due to their remote location. Distance from the DHO and other organizational covariates may have also captured dimensions of technical or behavioral facility characteristics. For instance, the distance from the DHO may capture farther facilities having limited infrastructure to support the technology, therefore lower likelihood of EIR use. (Indeed, distance to the DHO was collinear with urban/rural status in the Zambia model.) Similarly, in Tanzania, the increased likelihood of hospitals and health centers using the EIR may have also reflected that they were more likely to be connected to the electric grid.

The technical covariates we tested were not statistically significantly associated with weekly EIR use in the final model for either country or were excluded due to missing data. Primary power source was significantly associated with weekly EIR use in Tanzania in bivariate analysis. How-ever, facility type, public/private ownership, and the number of trained HCWs were associated with primary power missing data patterns, indicating the estimated association was biased. Primary power source was ultimately excluded from the final model due to missing data for Tanga region (Table 3). Although additional statistical methods would be needed to confirm the association between power supply and EIR use, the association observed in bivariate analysis was consistent with our hypothesis that facilities lacking a stable power source would be less likely to use the EIR. BID implementation staff in both countries had observed challenges with network availability, especially in rural facilities, or lack of data bundles that required some facilities to work in the EIR offline mode and later sync their data to the server.^38^

Finally, factors that were proxies for behavioral determinants—the number of HCWs initially trained and the number of weeks since EIR introduction—also emerged as important determinants. The number of weeks since EIR introduction was a strong predictor of decreased usage in both countries, with odds of system use declining by 1.9% each week in Tanzania and 12.4% in Zambia. This may have been due to waning HCW motivation or support to use the system over time. The more rapid decline in Zambia may have reflected the shorter window of ongoing support from the BID team post-EIR introduction compared to in Tanzania, the different intervention rollout strategies used,^25^ HCW perceptions of each country’s EIR system,^12^ or other factors. As mentioned, HCWs may have lost motivation to continue using the system if they perceived it as adding more work since they were still required to use the paper-based system for official reporting. Individual or facility recognition or incentives could have been powerful motivators to support behavior change but were applied inconsistently; failure to recognize consistent use combined with a lack of perceived data use may have contributed to waning motivation.

The number of weeks since EIR introduction was a strong predictor of decreased EIR use in both countries.

In Tanzania, the number of HCWs trained per facility during the EIR introduction period was significantly associated with increased odds of system use. This may suggest that facilities with more immunization HCWs had more bandwidth to use the system or perhaps that with more HCWs, they could have supported each other to continue system use. Across all facilities, additional support and accountability to encourage use of the EIR was needed to sustain use over time. In Zambia, the BID Initiative implementation phase ended shortly after EIR introduction in Southern Province was completed, so support significantly diminished. This may have, in part, explained the dramatic decline in system use over time. Demands on HCW time were numerous and without adequate incentives—whether financial or motivational—continuing a time-consuming practice may have been unreasonable to expect. Expectations of use from national or district leadership may have also played an important role in sustaining use.^39^

The MOHs in Tanzania and Zambia, in collaboration with the BID Initiative, continue to strengthen EIR usability. In Tanzania, there is ongoing work to identify additional facilities that are ready to transition to paperless reporting and to strengthen support for all facilities through a helpdesk line and district-level support. In Zambia, the MOH has focused on technical improvements to ZEIR, including fully integrating ZEIR with mVacc (an SMS-based platform to support community immunization awareness and access)^40^; transitioning data hosting to the MOH; refining district dashboards to allow for easy monitoring of facility data inputs; and reviewing potential syncing issues that may have impacted usage numbers reported in this study. In addition, an emphasis on engagement from local leaders at the subnational level has resulted in improved uptake of ZEIR among facilities. Like Tanzania, the Zambia MOH is assessing whether facilities can transition to paperless reporting to remove the burden of parallel systems. Finally, ZEIR is being used to monitor system use and data quality. The number of active users and the number of children present in the system each month are tracked as an indicator of use, and quality is assessed by comparing EIR records against those in paper registries.

This article presents one way of measuring use—based on the dates of data entered into the EIR—but there are other ways that system use could be measured. We chose this measure because it could be consistently measured across the available data from TImR and ZEIR. Using the dates attached to when services were delivered also allowed us to compare system use across facilities working in the online and offline modes; for example, if a facility entered a vaccine delivered on January 1 in offline mode and synced the data to the server on January 31, we counted that as system use for the first week of January. Alternatively, system use could be measured according to system logins or time stamps on when the data were entered or synced, and ideally would be built into automated reports for ongoing monitoring. We measured use in weekly increments to leverage the granularity of data, while acknowledging that most facilities provide immunization services at least once per week. As weekly use of the system stabilizes, it is important to assess data quality; for example, to measure internal consistency of the data by comparing the number of doses delivered over time or to measure external consistency by comparing the number of doses captured in the EIR to an external source, like survey data or paper-based child health cards or records.

As more countries move to introduce EIRs or other digital interventions, we recommend that they measure and monitor use of the system(s) among the intended users. New systems that aim to improve data timeliness, availability, or completeness will only be able to do so if they are used consistently as intended. Indicators for monitoring system use through metadata have been published by the World Health Organization and can be integrated into program plans.^31^ Measuring system use is important to: (1) inform interpretation of the data, since traditional reporting measures like coverage and dropout rates may be skewed depending on the completeness of data entry; and (2) inform programmatic decisions, such as targeting support to facilities with suboptimal use or determining when facilities are ready to transition away from traditional paper-based tools to using the EIR as their primary data collection and reporting tool.

New EIRs that aim to improve data timeliness, availability, or completeness will only be able to do so if they are used consistently as intended.

### Limitations

A key limitation of the analysis was data unavailability. Most available secondary data captured organizational and technical factors. We were limited in our ability to measure the impact of behavioral factors, including individual attitudes, skills, and motivation, which may be important in explaining differences in use across facilities. Also, some covariates had too much missing data to include in our models, such as primary power source in Tanzania. As a result, determinants included in the models may have been impacted by unmeasured confounding. For example, greater odds of EIR use at hospitals and health centers compared to dispensaries may have, in part, reflected greater access to electricity and internet. For the covariates we did include, most did not have information available on how they have changed over time, which may limit their ability to explain changes in use over time. For example, we captured the number of HCWs trained at the point of EIR introduction, but we do not have information on staff turnover. Finally, our analysis used all available EIR data for each region and given different EIR introduction dates, this resulted in a different number of data points per district. In Tanzania, trends may be driven by more data points from Arusha region compared to more limited follow-up time in regions that introduced the EIR later.^41^

## CONCLUSION

The results from this analysis add to our understanding of the organizational and behavioral factors associated with facility EIR use in Tanzania and Zambia. This analysis demonstrated greater EIR utilization among facilities at which HCWs reported into electronic systems only compared to parallel paper and electronic systems. In addition, it highlighted the importance of ongoing support for new digital interventions during and beyond the initial rollout, as demonstrated by greater EIR utilization at facilities with a greater number of HCWs trained in the intervention and at facilities closer to the DHO, and by decreased utilization over time. Strong district leadership and the mentorship and close supervision of HCWs have been considered essential to successful uptake and ongoing use of these systems.

The MOH and BID Initiative teams in each country should continue to collaboratively identify ways to improve EIR system use, such as continuing to transition facilities to paperless reporting, promoting the benefits of system use to HCWs, and making system use metrics available (e.g., through automated reports or dashboards) to empower stakeholders at all levels to monitor and support consistent use. System use metrics may need to be triangulated with other data sources (e.g., HCW surveys) or evaluation approaches to explain the observed trends in use.

Stakeholders introducing EIRs, or other digital health interventions, should consider providing additional support to more remote, lower-volume facilities and should develop plans from the start for when and how to transition to paperless reporting. However, these factors associated with system use may vary in different contexts. As EIRs are introduced in new contexts, we recommend building these types of analyses directly into the system. Moreover, if EIRs are designed to capture more granular information about facility characteristics and/or are linked to other routine health information systems, then additional data can be available to understand the different factors influencing system use.

## Notes

**Peer Reviewed**

**First published online:** August 19, 2020
